# Pharmacological Inhibition of MALT1 Ameliorates Autoimmune Pathogenesis and Can Be Uncoupled From Effects on Regulatory T-Cells

**DOI:** 10.3389/fimmu.2022.875320

**Published:** 2022-05-09

**Authors:** Subhabrata Biswas, Aditi Chalishazar, Ynes Helou, Joanna DiSpirito, Brian DeChristopher, Devin Chatterjee, Leidy Merselis, Benjamin Vincent, John G. Monroe, Dania Rabah, Andrew J. Long

**Affiliations:** ^1^ Immunology, Rheos Medicines, Cambridge, MA, United States; ^2^ Drug Discovery, Rheos Medicines, Cambridge, MA, United States; ^3^ Research and Development, Rheos Medicines, Cambridge, MA, United States

**Keywords:** MALT1 inhibitor, autoimmunity, regulatory T-cell, collagen-induced arthritis, IL-2

## Abstract

MALT1 forms part of a central signaling node downstream of immunoreceptor tyrosine-based activation motif (ITAM)-containing receptors, across a broad range of immune cell subsets, and regulates NF-κB driven transcriptional responses *via* dual scaffolding-protease activity. Allosteric inhibition of MALT1 activity has demonstrated benefit in animal models of inflammation. However, development of MALT1 inhibitors to treat autoimmune and inflammatory diseases (A&ID) has been hindered by reports linking MALT1 inhibition and genetic loss-of-function to reductions in regulatory T-cell (Treg) numbers and development of auto-inflammatory syndromes. Using an allosteric MALT1 inhibitor, we investigated the consequence of pharmacological inhibition of MALT1 on proinflammatory cells compared to regulatory T-cells. Consistent with its known role in ITAM-driven responses, MALT1 inhibition suppressed proinflammatory cytokine production from activated human T-cells and monocyte-derived macrophages, and attenuated B-cell proliferation. Oral administration of a MALT1 inhibitor reduced disease severity and synovial cytokine production in a rat collagen-induced arthritis model. Interestingly, reduction in splenic Treg numbers was less pronounced in the context of inflammation compared with naïve animals. Additionally, in the context of the disease model, we observed an uncoupling of anti-inflammatory effects of MALT1 inhibition from Treg reduction, with lower systemic concentrations of inhibitor needed to reduce disease severity compared to that required to reduce Treg numbers. MALT1 inhibition did not affect suppressive function of human Tregs *in vitro*. These data indicate that anti-inflammatory efficacy can be achieved with MALT1 inhibition without impacting the number or function of Tregs, further supporting the potential of MALT1 inhibition in the treatment of autoimmune disease.

## Introduction

Autoimmune and inflammatory diseases (A&ID) such as rheumatoid arthritis, systemic lupus erythematosus, and Crohn’s disease are among the most prevalent chronic illnesses in the United States, collectively affecting 5-8% of the total population. These diseases bring about immense suffering to patients greatly reducing quality of life with a high morbidity and mortality burden and significant socioeconomic impacts (1, 2). Despite rapid advances in our understanding of the pathophysiology of A&ID, and the continued development of novel targeted therapies, considerable unmet need remains due to lack of complete and sustained disease remission and otherwise inadequate response to treatment by a large fraction of patients. The latter is likely due to the inherent mechanistic heterogeneity of these diseases and the involvement of multiple immune and non-immune cell types.

Mucosa-associated lymphoid tissue lymphoma translocation protein 1 (MALT1) forms part of the intracellular CBM (CARMA/CARD-BCL10-MALT1) signaling complex downstream of receptors containing ITAMs as well as a subset of G-protein-coupled receptors (GPCRs) and receptor tyrosine kinases found in both hematopoietic and non-hematopoietic cell types (3, 4). Following receptor activation, the CBM complex activates downstream signaling events *via* the scaffolding and protease functions of MALT1 that regulates key cellular processes, including lymphocyte development and activation of cells of the innate and adaptive immune system. MALT1 has been studied extensively in activated lymphoid cells, particularly in the context of T-cell and B-cell receptor (TCR/BCR) activation. A central role of MALT1 in the amplification of ITAM-driven inflammatory responses across multiple immune cell types, including immune complex activation of myeloid cells (4), makes it a compelling target for the treatment of A&ID. Data from pre-clinical models also support the validity of MALT1 as a drug target. Genetic inactivation or pharmacological inhibition of MALT1 protects against experimental autoimmune encephalomyelitis (EAE), arthritis, psoriasis, and colitis (5–11). The data presented in this paper confirm the pharmacologic activity of MALT1 inhibition both *in vitro* and in *in vivo* animal models of A&ID.

Although promising, the therapeutic potential of targeting MALT1 has been hindered by concerns regarding the impact of MALT inhibition on Treg homeostasis. Genetic studies in mice, and pharmacologic inhibition in multiple species, have indicated that MALT1 signaling is critical for maintaining immune homeostasis (12). Treg-restricted perturbation of MALT1 protease activity results in a severe multi-organ autoimmune phenotype, including systemic and neuroinflammatory disorders, resembling an IPEX-like syndrome (immune dysregulation, polyendocrinopathy, enteropathy, X-linked) (13). Additionally, preclinical safety assessment of an allosteric small molecule inhibitor of MALT1 (MLT-943) in naïve rats and dogs demonstrated Treg loss and progressive lymphadenopathy, lymphocytic infiltration of multiple tissues and gastrointestinal toxicity following prolonged dosing (14). In contrast, a recent study using conditional inactivation of MALT1 in adult mice did not report systemic inflammation, despite reduced Treg numbers, indicating that the relationship between MALT1 activity, Treg reduction and subsequent systemic inflammation remains uncertain (15). Similarly, an allosteric small molecule inhibitor of MALT1 was recently described which maintained T-effector cell/Treg homeostasis *in vivo* (16). Together, these studies point to a lack of clarity about the impact of MALT1 inhibition on Treg function. Furthermore, the data suggest that targeting MALT1 could be an effective therapeutic option if inhibition of immune cell effector functions could be uncoupled from effects on Treg survival and function.

In the current study, we provide evidence that MALT1 inhibition has broad effects on inflammatory mechanisms both *in vitro* and *in vivo*. Importantly, the efficacious, anti-inflammatory effects of MALT1 inhibition were shown to be uncoupled from decreases in Treg numbers. In addition, reduction in Treg numbers as a consequence of MALT1 inhibition was less pronounced in the context of inflammation when compared to healthy subjects, and furthermore, there was no direct effect of MALT1 inhibition on Treg suppressive capacity or IL-2 signaling. Combined, these data provide proof-of-concept for effectively targeting MALT1 in inflammatory diseases without deleterious impact on Tregs.

## Materials And Methods

### Compound Synthesis and Characterization

The primary compound used in this study (designated as MALT1i) is a previously described allosteric small molecule inhibitor of MALT1 and was synthesized as per the published protocol (14). Biochemical characterization of the compound was performed *via* measurement of enzymatic activity against MALT1, as described in the **Supplementary Material**. Relative and temporal effects of the compound across MALT1 scaffolding and paracaspase/protease functions was determined by immunoblotting of cell lysates prepared from activated primary human CD4^+^ T-cells treated with MALT1i (**Supplementary Material**).

### Jurkat IL-2 Assay

Jurkat human T-cell line (ATCC, clone E6.1) was exposed to a range of MALT1i concentrations and assessed for viability and inhibition of cytokine expression following cell activation. Cells were cultured in RPMI/10% FBS (Thermofisher, Waltham, MA) and maintained under a concentration of 3 x 10^6^ cells/ml. MALT1i at different concentrations were stamped by ECHO onto 384-well plates (PerkinElmer, Waltham, MA) following which cells were plated in fresh media and incubated for 30 min before stimulation with soluble α-CD3/-CD28/-CD2 (ImmunoCult, Stemcell Technologies, Vancouver, Canada) for 24 h. Supernatants were collected and processed immediately for cytokine analysis or stored at -80°C. To assess viability of cells treated with compound, cells were lysed with CTG reagent (Promega, Madison, WI), and measured by luminometer.

### Primary Human Immune Cell Assays

Total CD4^+^ T-cells were isolated from human donor peripheral blood mononuclear cells (PBMC) using an EasySep kit (Stemcell) and pre-incubated with MALT1i (0-5 µM) for 30 min at 37°C, 5% CO_2_ prior to stimulation with ImmunoCult Human CD3/CD28 T-cell activator (Stemcell) per manufacturer’s protocol.

Memory CD4^+^ T-cells were isolated from human donor PBMCs using an EasySep kit (Stemcell) and rested overnight in complete RPMI media (Thermofisher) supplemented with 10% FBS (Atlanta Biologics, Flowery Branch, GA), 10 mM HEPES, 2 mM GlutaMAX, 1 mM Na Pyruvate, and 1X MEM non-essential amino acids (Thermofisher). Cells were treated with varying concentrations of MALT1i (0-30 µM) for 30 min at 37°C, 5% CO_2_ prior to stimulation with ImmunoCult Human CD3/CD28/CD2 T-cell activator (Stemcell).

Monocytes were isolated from human donors using an EasySep kit (Stemcell) and were differentiated into macrophages in RPMI-1640 medium, supplemented with 10% FBS, 2 mM GlutaMAX, 100 IU/ml penicillin and 100 IU/ml streptomycin (Thermofisher) for 6 days in petri dishes in the presence of 50 ng/ml M-CSF (Peprotech, Cranbury, NJ). After differentiation, cells were harvested with Accutase solution (Stemcell) and incubated for 2 h at 37°C, 5% CO_2_ in 96-well plates. Cells were treated with varying ratios of MALT1i (0-30 µM) for 30 min at 37°C prior to stimulation with 50 μg/ml depleted-zymosan (Invivogen,, San Diego, CA) or IgG Immune Complex. For IgG Immune Complex preparation, 92 μg/ml of Anti-Human IgG (Jackson Immunoresearch, West Grove, PA) was gently laid on top of 10 μg/ml of Human IgG (Bio-Rad, Hercules, CA) and was incubated for 1 h at 37°C, 5% CO_2_ prior to stimulating the cells as mentioned earlier.

Freshly isolated total CD19^+^ B-cells (Stemcell) from human PBMCs were labeled with 2 μM Cell Trace Violet (ThermoFisher) and incubated for 5 min on a Nutator. Cells were then washed, plated and treated with MALT1i (0-1 µM) for 30 min at 37°C, 5% CO_2_ prior to stimulation for 4 days with 50 μg/ml soluble F(ab’)2 anti-human IgA+IgG+IgM (H+L) (Jackson Immunoresearch, West Grove, PA), 100 ng/ml soluble recombinant CD40L (TNFSF5) (ThermoFisher). Proliferation was assessed by flow cytometry.

### Whole Blood Potency Assay

Heparinized human whole blood was obtained from healthy donors (Research Blood Components, Watertown, MA). Blood samples were diluted 1:1 with RPMI 1640 (Thermofisher Scientific, Waltham, MA) and aliquoted into 96-well plates with a final volume of 100 μl. Samples were treated with different concentrations of MALT1i from 0-30 μM in a volume of 10 μl and incubated for 30 min at 37°C following which stimulation of the treated blood was performed with 1 μg/ml each of α-CD3 (clone UCHT1, Thermofisher) and α-CD28 (clone CD28.2, Thermofisher) in a volume of 10 μl and incubated for 48 h at 37°C + 5% CO_2_. Plates were spun 470 xg for 10 min and ~ 40 μl of supernatant (plasma) separated from each well and stored at -80°C prior to cytokine analysis.

Heparinized whole blood was obtained from naïve 8–10-week-old Sprague Dawley rats (Charles River Laboratories, Wilmington, MA). Treatments of 1:1 diluted blood samples were performed similarly as with the human whole blood. Following incubation with different concentrations of MALT1i from 0-30 μM, the blood samples were stimulated with 10 μl of phorbol 12-myristate 13-acetate (PMA)/Ionomycin (Sigma, St. Louis, MO) at concentrations of 25 ng/ml and 1 μg/ml, respectively and as previously described (17). Plates were then incubated for 6 h at 37°C + 5% CO_2_, spun down and the supernatant separated as described for the human whole blood assay. Samples were stored at -80°C prior to cytokine analysis.

### Rat Collagen-Induced Arthritis Model

Adult female Lewis rats (Charles River) with body weights between 180-200 g were immunized subcutaneously with bovine type II collagen (Chondrex, Woodinville, WA)/Incomplete Freund’s Adjuvant (Sigma) emulsion prepared per manufacturer’s protocol (Chondrex) on day 0 and day 7. MALT1i doses (0.3 – 10 mg/kg) for oral administration were prepared by suspending the compound in 0.5% Na-carboxymethylcellulose/0.5% Tween-80 in water (vehicle). For prophylactic treatment animals were dosed *via* oral gavage on day 0 prior to immunization with collagen and continued once daily (q.d.) for four weeks. For therapeutic treatment, animals were randomized per clinical disease scoring on day 12 and q.d. dosing of compounds *via* oral gavage was carried out for two weeks. Vehicle treated and naïve animals were used as controls. Clinical score and joint swelling (hind limb volume) were measured on day 0, before the first dosing and then 3 times a week until the end of the study. Body weight measurements were performed thrice weekly to assess compound tolerability. Criteria (on a scale of 0-4 per limb) were as follows: 0, No evidence of erythema and swelling; 1, Erythema and mild swelling confined to the mid-foot (tarsals) or ankle joint; 2, Erythema and mild swelling extending from the ankle to the mid-foot; 3, Erythema and moderate swelling extending from the ankle to the metatarsal joints; 4, Erythema and severe swelling encompassing the ankle, foot, and digits. Following 2-3 weeks of dosing, representative animals were bled at different time intervals over a 0-24 h time-period to assess compound exposure in the plasma *via* LC/MS.

For isolation of synovial fluid prior to study termination, animals were anesthetized and the skin on the hind limbs were separated to ensure exposure of knee joint. A small incision was made near the top of the knee joint and the articular cavity was rinsed 2 times with 60 μl phosphate-buffered saline (PBS) and ~50-60 μl of synovial fluid was collected. The synovial fluid samples were spun down, and the supernatant stored at -80°C prior to cytokine analysis. At study termination, plasma was prepared from whole blood and stored at -80°C prior to cytokine analysis and measurement of total and anti-collagen IgG. Single cell suspensions were prepared from harvested spleens following standard methods. Treg frequencies were measured by flow cytometry-based immunophenotyping of freshly isolated cells.

### Immunophenotyping

Harvested spleens were homogenized in cold PBS and cellular debris removed by passing through a 70 μM cell strainer. The cell suspension was centrifuged at 300 xg for 5 min at 4°C and the pellet was treated with 1X RBC lysis buffer per manufacturer’s protocol (Thermofisher). The washed cell pellet was resuspended for a final concentration of 5-10 × 10^6^ cells/ml and 100 μl of cell suspension was processed for immunophenotyping. Briefly, cells were first stained with a Fixable Live-Dead dye (BD Biosciences, Franklin Park, NJ) followed by FcR blocking with a rat anti-CD32 antibody (BD) per manufacturer’s protocol. Cells were then stained with a panel of fluorescently labeled antibodies (BD, unless otherwise mentioned) targeting rat immune cell surface markers, that included CD45 (clone OX-1, pan lymphocyte marker), CD3 (clone 1F4,T-cell), CD45RA (clone OX-33, B-cell), CD11b/c (clone OX-42, monocyte/macrophages), CD4 (clone OX-35, helper T-cells), CD8 (clone OX-8, cytotoxic T-cells), CD25 (clone OX-39, high affinity IL-2 receptor α), for 30 min on ice. Cells were washed with cold FACS wash buffer (BD) and fixed with 1XFoxP3/Transcription Factor Fixation Buffer (Thermofisher) for overnight at 4°C. Cells were subsequently permeabilized by washing with 1XFoxP3/Transcription Factor Permeabilization and Wash Buffer (Thermofisher) followed by staining for intracellular FoxP3 (clone 150D, Biolegend, San Diego, CA), a canonical Treg marker, for 30 min. Cells were washed 2-3X with the permeabilization and wash buffer as mentioned earlier, and finally resuspended in FACS wash buffer. Samples were acquired on a BD LSRFortessa flow cytometer and data analyzed using the FlowJo software (BD).

### Cytokine Analysis

All cytokine measurements were performed using commercially available kits and as per manufacturer’s protocol. Supernatants from human whole blood and cellular assays were quantified using the human Proinflammatory Panel 1 (human) kit on a Sector Imager 6000 reader (Meso Scale Discovery, Rockville, MD). IL-17A was quantified using the V-Plex Human IL-17A kit on a Sector Imager 6000 reader (MSD). Supernatants from rat whole blood assay were analyzed for IL-2 levels using the rat IL-2 Duoset ELISA kit (R&D Systems, Minneapolis, MN). Plasma and synovial samples harvested from the rat CIA study were quantified for proinflammatory cytokines using the rat V-PLEX Proinflammatory Panel 2 kit (MSD).

### Anti-Collagen Antibody and Total IgG

Anti-collagen antibodies in rat CIA plasma were analyzed using the Rat anti-bovine Type II Collagen IgG Antibody ELISA Kit (Chondrex). The assay was performed according to the manufacturer’s protocol, with samples diluted 1:100,000. Total IgG antibodies in rat plasma were analyzed using the Rat total IgG Uncoated ELISA Kit (Invitrogen, Waltham, MA). The assay was performed according to the manufacturer’s protocol, with samples diluted 1:400,000.

### Treg Isolation, Expansion, and *In Vitro* Suppression Assay

Tregs were isolated from human donor PBMCs using the Human CD4^+^CD127^low^CD25^+^ Regulatory T-Cell Isolation Kit (Stemcell) and expanded in culture as previously described (18). Briefly, Tregs were activated with Human T-Activator CD3/CD28 Dynabeads (Thermofisher) at a 1:1 bead to cell ratio in complete RPMI media supplemented with 10% FBS, 10 mM HEPES, 2 mM GlutaMAX, 1 mM Na-Pyruvate, and 1X MEM non-essential amino acids (Thermofisher). After 2 days in culture, the culture volume was doubled, and IL-2 was added at a final concentration of 300 IU (Peprotech). At days 5 and 7, cells were expanded in the presence of 300 IU IL-2. On day 9, cells were restimulated with Dynabeads at a 1:1 bead to cell ratio. On day 13, Tregs were harvested, and beads were magnetically removed for downstream Treg suppression assays.

For the *in vitro* Treg suppression assay, naïve CD4^+^ T-cell proliferation was assessed by measuring Cell Trace Violet (CTV) (Thermofisher) dilution in the presence of varying ratios of autologous Tregs. In brief, naïve CD4^+^ T-cells were isolated from human donor PBMCs using the Human Naïve CD4^+^ T-cell Isolation Kit (Stemcell) and labeled with CTV according to the manufacturer’s protocol. Naïve CD4 T^+^-cells were activated with Human T-Activator CD3/CD28 Dynabeads at a 1:8 bead to cell ratio for 3 days in the presence of varying ratios of MALT1i-treated Tregs in complete RPMI. Cells were then processed for flow analysis of proliferation. Briefly, cells were first stained with a Fixable Live-Dead dye (BD) and then stained with the following panel of fluorescently labeled antibodies (Biolegend, unless otherwise mentioned): CD4 (clone RPA-T4) and CD25 (clone M-A251), for 20 min at 4°C. Cells were subsequently stained for intracellular FoxP3 (clone 236A/E7, Invitrogen) using the Foxp3 Transcription Factor Staining Buffer Set (eBiosciences) according to the manufacturer’s instructions. Cells were analyzed on a BD LSRFortessa flow cytometer. Percent suppression was calculated using the following formula: % suppression = ((% naïve T-cell proliferating) – (% naïve T-cell + Treg proliferating))/(% naïve T-cell proliferating) x 100.

### pSTAT5 Measurement in Tregs

Purified Tregs were expanded as indicated above. On day 12, cells were harvested and incubated with MALT1i at various concentrations for 18 h in serum-free RPMI at 37°C, 5% CO_2_. Tregs were then stimulated with 25 IU IL-2 (Peprotech) for 15 min, followed by fixation with 2% paraformaldehyde. Cells were then permeabilized with 90% methanol and stained with an anti-pSTAT5 antibody (Y694, clone 47, BD) at a 1:100 dilution. Levels of pSTAT5 were analyzed on a BD LSRFortessa flow cytometer.

### Statistics

All statistical calculations were performed using Graphpad Prism 9.2.0. Datapoints from most readouts, e.g., cytokine expression, cell proliferation, disease scores, antibody levels, etc. were expressed as mean ± standard error of mean (S.E.M). Significant differences (*p*<0.05) across unpaired observations were calculated using One-way ANOVA and corrected for multiple comparisons using Dunnett’s test. Comparison between two groups (e.g., healthy vs arthritic animals) were performed using a Mann-Whitney test. Cytokine expression data in treatment groups were expressed as a percent of DMSO control normalized to 100% activity. Potency measurements, IC_50_ and IC_90_ values, were performed using a 4-parameter fit in GraphPad Prism. Pharmacokinetic-pharmacodynamic (PK-PD) correlations were expressed by similarly fitting plasma concentrations of MALT1i over a 24 h period since last dose (AUC_0-24h_) from individual subjects to the corresponding effects observed with regards to reduction in clinical scores and/or splenic Tregs.

## Results

### Pharmacological Modulation of MALT1 Dampens Multiple Immune Effector Functions in Primary Human Cells *In Vitro*

**Figure 1:**
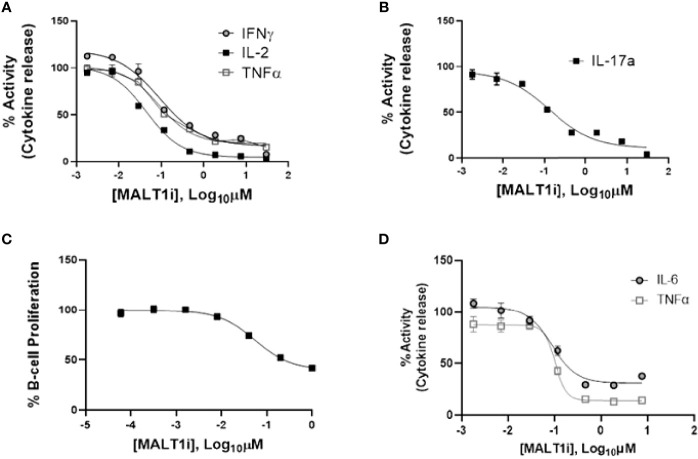
Inhibition of MALT1 with an allosteric small molecule inhibitor (MALT1i) suppresses adaptive and innate immune effector mechanisms *in vitro*. **(A)** Suppression of proinflammatory cytokines - IFNγ, IL-2 and TNFα from CD45RO^+^ memory T-cells and activated *via* cross-linking of the T-cell receptor (α-CD3/-CD28/-CD2) for 24 h in the presence of increasing concentrations of MALT1 inhibitor. **(B)** Suppression of IL-17A from CD45RO^+^ memory T-cells activated *via* cross-linking of the T-cell receptor + co-stimulation (α-CD3/-CD28/-CD2) for 48 h in the presence of MALT1i. **(C)** Human B-cell proliferation induced *via* IgM/CD40L stimulation was attenuated by MALT1i. **(D)** Production of IL-6 and TNFα from immune-complex stimulated human monocyte derived macrophages was inhibited with MALT1i. See **Table 1** for potency values from individual cellular assays.

We evaluated the impact of MALT1i (**Supplementary Figure 1A**) in inflammatory processes associated with adaptive and innate immune cells activated *via* stimulation of distinct ITAM-containing immunoreceptors (**Figure 1** and **Table 1**). MALT1i inhibited the release of proinflammatory cytokines (IFNγ, IL-2 and TNFα) and IL-17A from CD45RO^+^ memory T-cells activated *in vitro* by α-CD3/-CD28/-CD2 (**Figures 1A, B**, respectively) in a dose-dependent manner. MALT1i also attenuated B-cell proliferation stimulated *via* co-crosslinking of BCR/CD40L with anti-human IgA/IgG/IgM and recombinant CD40L (**Figure 1C**). In myeloid cells, MALT1i effectively suppressed proinflammatory cytokine expression from human monocyte-derived macrophages differentiated *in vitro* and stimulated *via* FcγR with anti-human IgG/human IgG-derived immune complexes (**Figure 1D** and **Table 1**). Importantly, macrophages stimulated with bacterial lipopolysaccharide (LPS), which does not signal through an ITAM-dependent mechanism, were insensitive to MALT1i (data not shown), consistent with previous observations (19). By contrast, macrophages stimulated with depleted zymosan, which signals through the ITAM-dependent Dectin-1 receptor, were sensitive to MALT1 inhibition (**Table 1**). Taken together, these data show that inhibition of MALT1 with an allosteric inhibitor can selectively suppress ITAM-driven autoimmune inflammatory processes by multiple immune cell types in a stimulus-restricted manner.

**Table 1:** Determination of MALT1i potency in *in vitro* cellular assays.

Cells/(stimulation)	Readout	IC_50_ (μM)	IC_90_ (μM)	Effect size (% Max. inhibition)
*Jurkat-T cells *(α−CD3/-CD28/-CD2)	IL-2	0.03 ± 0.002	0.20 ± 0.01	>95
*CD4^+^CD45RO^+^ Memory T- cells* (α−CD3/-CD28/-CD2)	IL-2	0.05 ± 0.01	0.31 ± 0.07	>90
	IFNγ	0.09 ± 0.02	1.49 ± 0.52	~80
	TNFα	0.09 ± 0.04	0.80 ± 0.16	~85
*CD4^+^ Total T- cells *(α−CD3/-CD28)	IL-2	0.03 ± 0.009	0.17 ± 0.003	>95
	IFNγ	0.13 ± 0.06	0.37 ± 0.08	>95
	TNFα	0.05 ± 0.008	0.09 ± 0.002	~85
*CD4^+^CD45RO^+^ Memory T- cells *(α−CD3/-CD28/-CD2)	IL-17	0.13 ± 0.02	1.71 ± 0.36	>90
Total B cells (*BCR/CD40L*)	Proliferation	0.06 ± 0.004	0.45 ± 0.13	~60
Monocytes → Macrophages (*depleted zymosan*)	TNFα	0.07 ± 0.02	0.32 ± 0.11	>75
	IL-6	0.06 ± 0.07	0.25 ± 0.21	>60
Monocytes → Macrophages *(αIgG + IgG, immune-complex)*	TNFα	0.09 ± 0.01	0.35 ± 0.16	~74
	IL-6	0.05 ± 0.03	0.28 ± 0.07	>75

BCR, B-cell receptor. Values plotted are Mean ± S.E.M from assays performed twice with 1-3 donors. Potency values determined using Graphpad Prism software.

### MALT1 Inhibition Blocks Cytokine Expression in Stimulated Whole Blood From Multiple Species

As reported earlier, MALT1i resulted in the reduction of peripheral Treg levels following chronic administration to healthy animals (rat, mouse and dog) (14). In order to understand the dose-dependent relationship between MALT1i efficacy and Treg effects, we first developed a surrogate measure of *in vivo* target engagement using a whole blood assay. In rat and human whole blood, spike-in of MALT1i was followed by TCR stimulation (a MALT1-dependent pathway) and measurement of cytokine secretion. This assay takes into account plasma protein binding and enables understanding of the potency of the inhibitor in a complex mixture of cells. As shown in **Table 2**, pretreatment of human and rat whole blood with MALT1i and subsequent co-stimulation of the TCR with antibodies directed to CD3 and CD28, or PMA/ionomycin (surrogate ITAM signaling), respectively, resulted in suppression of proinflammatory cytokine production. In response to CD3/CD28 signaling, several cytokines were impacted (IL-2, IFNγ and TNFα) and IL-2 was most sensitive to MALT1 inhibition. The IL-2 response in rat whole blood stimulated with PMA/ionomycin showed a similar IC_50_ compared to anti-CD3/-CD28 stimulation.

**Table 2:** Determination of MALT1i potency in whole blood.

Stimulus	Cytokine	IC_50_ (μM)	IC_90_ (μM)	Effect Size (% Max. inhibition)
***Human*** α−CD3/-CD28	IL-2	0.27 ± 0.01	1.39 ± 0.60	>99
	IFNγ	0.79 ± 0.13	5.38 ± 0.76	
	TNFα	0.59 ± 0.19	3.83 ± 0.38	
***Rat*** PMA/ionomycin	IL-2	0.18 ± 0.02	1.90 ± 0.29 (est.)	70

PMA, phorbol 12-myristate 13-acetate; est., estimated. Values plotted are Mean ± S.E.M from assays performed twice with 1-3 donors/animals. Potency values determined using Graphpad Prism software.

We next determined the relationship between the cytokine PD effect and target engagement by measuring the dose-dependent effects of MALT1i on proteolytic cleavage of a known MALT1 substrate. Using immunoblotting, cleavage of the MALT1 substrate HOIL-1 was monitored across a range of MALT1i concentrations, encompassing 50-90% of target coverage as determined using the human whole blood assay. At a compound concentration equivalent to human whole blood IL-2 IC_50_, protease function was impacted by >80% (**Supplementary Figure 1B**).

### Treatment With MALT1i Ameliorates Disease Pathogenesis in Rat Collagen-Induced Arthritis

**Figure 2:**
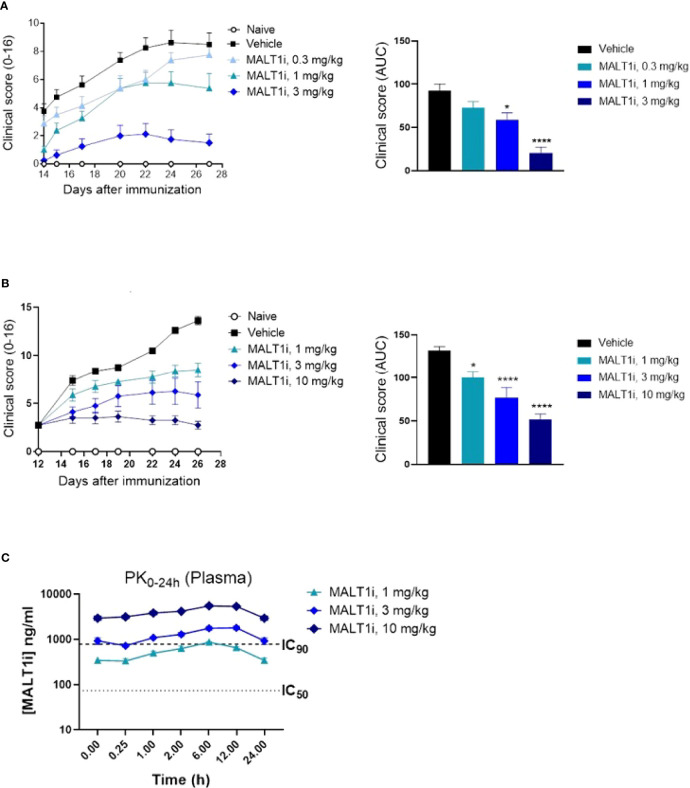
Oral administration of MALT1i resulted in a dose-dependent reduction in disease severity and progression in a rat model of collagen-induced arthritis (CIA). **(A)** Clinical scores were reduced in rats (n = 8/group) treated with MALT1i prior to immunization with collagen (days 0, 7) with indicated doses once daily for four weeks (prophylactic, left). Clinical scores measured three times per week starting from day 14 are plotted (mean ± S.E.M) for each group. Total disease burden over time is represented by plotting the calculated area under curve (AUC) across the treatment groups (right). **(B)** Alternatively, animals were immunized with collagen as indicated, randomized on day 12 and treated with MALT1i for two weeks (therapeutic, right). Clinical scores measured three times per week are plotted (mean ± S.E.M) for each group. Total disease burden over time is represented by plotting the calculated area under curve (AUC) across the treatment groups (right). Significant difference from the vehicle treated group was calculated *via* One-way ANOVA using Graphpad Prism, *p < 0.05, ****p < 0.0001. **(C)** Plasma concentration of MALT1i was measured between 0-24 h following last dose. Rat whole blood potency (IC_50_ and IC_90_, see **Table 1**) values indicated as dotted lines. *x-axis* not to scale.

Many of the immune effector mechanisms described above are implicated in autoimmune disease processes, hence we next assessed the effect of MALTi administration in a rat CIA model. MALT1i was formulated using 0.5% Na-carboxymethylcellulose/0.5% Tween-80 in water and administered either prophylactically (days 0-27) or therapeutically when the disease is established (days 12-25) at 3 mg/kg and 10 mg/kg. Oral administration of MALT1i demonstrated a dose-dependent decrease in disease activity scores in both prophylactic (**Figure 2A**) and therapeutic (**Figure 2B**) dosing regimens, as shown by reduction in clinical score over time and lower overall disease burden. These data are consistent with previous results in rat CIA by Martin et al., using the same compound, same vehicle, and dose levels in therapeutic regimen (14). No drug concentrations were reported from the rat CIA data in the published work to directly compare PK/efficacy correlation in the two studies. Taken together, these data are consistent with the effect of MALT1i on T and B-cell responses (disease initiation) and FcγR driven myeloid cell responses that are believed to be important for disease maintenance (20). Analysis of MALT1i levels in the plasma of compound-treated animals indicated that MALT1i is efficacious at concentrations that result in 90% target coverage (rat whole blood IC_90_, **Table 2**) for ≥24 h following dosing (**Figure 2C**).

**Figure 3:**
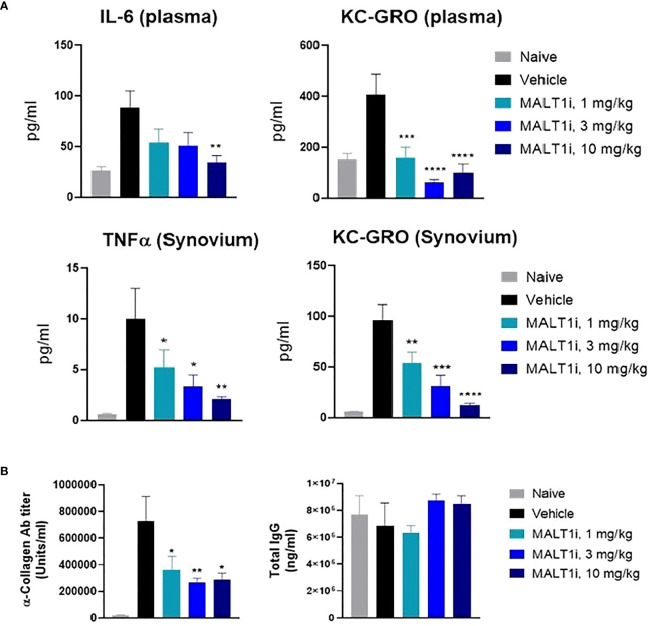
Therapeutic treatment with MALT1i suppresses proinflammatory cytokine and auto-antibody production in rat model of collagen induced arthritis (CIA). **(A)** Proinflammatory cytokines in the plasma (top) and in synovium (bottom) harvested from whole blood and knee joints, respectively, were measured at study termination. Cytokines that showed significant change in MALT1i treated groups compared to vehicle are plotted. Significant difference from the vehicle treated group was calculated *via* One-way ANOVA using Graphpad Prism, *p < 0.05, **p < 0.01, ***p < 0.001, ****p < 0.0001. **(B)** MALT1 inhibition resulted in a dose dependent reduction in antigen specific autoantibody (α-collagen IgG) level while sparing total IgG antibody titers. Statistical analysis performed using One-way ANOVA (*p < 0.05, **p < 0.01).

The effector phase in the rat CIA model is characterized by systemic inflammatory responses and by inflammation in the joints caused by neutrophil accumulation and deposition of antigen-antibody immune complexes, with resultant activation of the complement cascade. Proinflammatory cytokines (e.g., TNFα, IL-1β) secreted by activated macrophages are also implicated in sustaining inflammation (20). At study termination, the highest dose of MALT1i significantly reduced IL-6 levels in the plasma. Significant inhibition of KC-GRO, a neutrophil chemoattractant produced by activated macrophages, was also observed in both plasma and synovial fluid. Significant reduction of TNFα in the synovial fluid was also observed at all doses (**Figure 3A**). Furthermore, analysis of the plasma for anti-collagen antibodies, which are implicated in immune-complex formation, showed significant reduction with MALT1i treatment (**Figure 3B**). Importantly, there was no effect on total IgG levels across all treatment groups when compared with naïve animals, suggesting that this level of target coverage does not lead to complete immunosuppression, although more systemic effects will need to be defined in subsequent studies. These data indicate that maintaining a systemic MALT1i exposure equivalent to IC_90_ coverage over the full 24 h period is not necessary to suppress known disease drivers of chronic inflammation and suggest a lack of broad immunosuppression.

### Dose-Dependent Relationship Between Efficacy and Treg Reduction Points to an Uncoupling of Disease Amelioration and Treg Reduction

Given that Treg reductions have been observed with genetically deleted MALT1 (12) or with high doses of MALT1 inhibitor (14), we set out to determine if efficacy could be achieved without impacting the Treg compartment following MALT1i administration in the rat CIA model.

**Figure 4:**
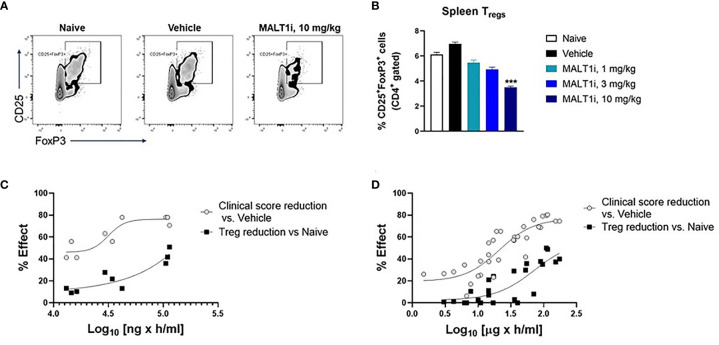
MALT1 inhibition leads to a dose-related uncoupling of efficacy and Treg reduction. **(A)** Representative flow cytometry plots showing frequency of CD4^+^ gated CD25^+^FoxP3 ^+^ Tregs in rat splenocytes at study termination. **(B)** Frequency of splenic Tregs was significantly reduced at the highest dose of MALT1 inhibition. **(C)** Dose-response (PK-PD) correlation for individual animals in MALT1i treated groups is shown. Plasma MALT1i concentrations expressed as AUC_0-24h_ (*x-axis*) is plotted against corresponding clinical scores (percent reduction vs. vehicle) and frequency of splenic Tregs (percent reduction vs. naïve) (*y-axis*). Curve-fitting for AUC/clinical score and AUC/Treg relationships were performed using Graphpad Prism to show dose-related uncoupling of efficacy and Treg reduction. **(D)** Compiled exposure response data from 4 distinct MALT1 inhibitors show consistent dose-related uncoupling of efficacy and Treg reduction. Statistical analyses performed using One-way ANOVA (***p < 0.001).

**Figure 4A** depicts representative flow cytometry plots and subsequent quantification of the frequency (**Figure 4B**) of splenic CD4^+^CD25^+^FoxP3^+^ Tregs isolated from CIA rats after therapeutic treatment with MALT1i. We observed statistically significant decreases in Treg numbers at the highest dose of inhibitor. To define the drug exposure: efficacy: Treg relationship, we paired these Treg measurements against measurements of disease score and compound concentrations. An exposure-response analysis revealed an uncoupling of efficacy in CIA from Treg reduction (**Figure 4C**). Specifically, we observed that a drug concentration AUC of 31,500 ng × h/ml was necessary to achieve 50% effect on disease score while a drug concentration AUC of 155,000 ng × h/ml was necessary to reduce Treg numbers by 50% compared to naïve animals (**Figure 4C**). To confirm that this uncoupling of efficacy from Treg reduction was not a function of the specific compound tested, we analyzed exposure-responses from four distinct MALT1 inhibitors (Rheos proprietary compounds). We consistently observed that the drug concentrations required to achieve a significant effect on efficacy were 3-5X lower than the drug concentrations required for reduction in Treg numbers (**Figure 4D**), suggesting that uncoupling of efficacy in CIA from reductions in Tregs is a generalizable feature of allosteric inhibition of MALT1.

In addition to Treg levels, immunophenotyping of other splenic immune cell subsets was performed. We did not observe significant effect of MALT1 inhibition on CD4 and CD8 subsets as well as monocyte/macrophage frequencies compared to the vehicle control (**Supplementary Figure 2**).

### MALT1 Inhibitor-Induced Treg Reduction Is Less Pronounced in Diseased Animals Than in Naïve Animals

**Figure 5:**
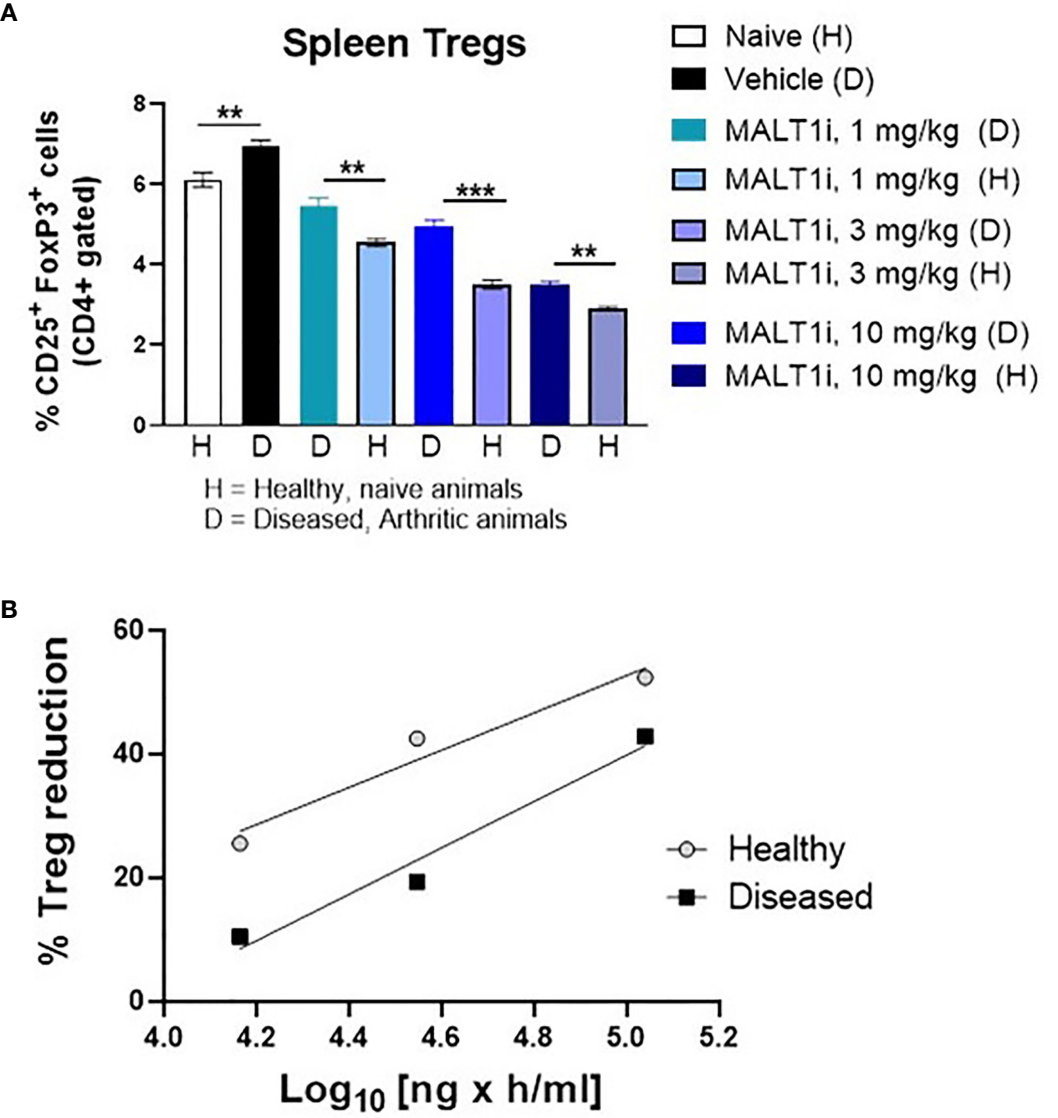
Tregs in diseased animals are less sensitive to MALT1i than in healthy animals. **(A)** Naïve, healthy animals were treated with MALT1i in parallel with the animals in the rat CIA study for two weeks. Frequencies of splenic Tregs were compared between healthy (H) and diseased (D) animals by flow cytometry (n = 8/group). Significance between similar dosing groups in healthy and diseased animals was calculated using Mann Whitney Test using Graphpad Prism software, **p < 0.01, ***p < 0.001, **(B)** Average Treg frequencies (compared to the naïve untreated group) from each MALT1i dosing group were plotted against the corresponding mean pharmacokinetic (PK) data expressed as AUC. PK was calculated from diseased animal plasma only and values were extrapolated to the healthy control animals.

Previous studies with healthy animals observed a rapid partial reduction in peripheral Treg cells following chronic dosing with MALT1i. This occurred in multiple species, including rats (14). Reasoning that an inflammatory state in the collagen immunized rats could alter the effect of MALT1 inhibition on the Treg compartment, we assessed the effect of MALT1i treatment in both naïve rats, and in CIA rats with active disease. To match the dosing paradigm of the CIA model, naïve rats were treated for 14 days with MALT1i, and Tregs were measured contemporaneously in naïve and CIA rats treated with the same three doses of MALT1i. We observed that Treg numbers were slightly elevated in the context of disease and that reductions in Treg numbers in the spleens of MALT1i-treated naive rats were significantly more reduced at any given dose than Treg numbers from spleens of rats with active CIA (**Figure 5A**). This observation was further confirmed with an exposure-response analysis, which showed that at similar plasma drug concentrations, the effect of Treg reduction was more pronounced in naïve animals compared with diseased animals (**Figure 5B**).

### MALT1 Inhibition Does Not Directly Block Treg Suppressive Function *In Vitro*

**Figure 6:**
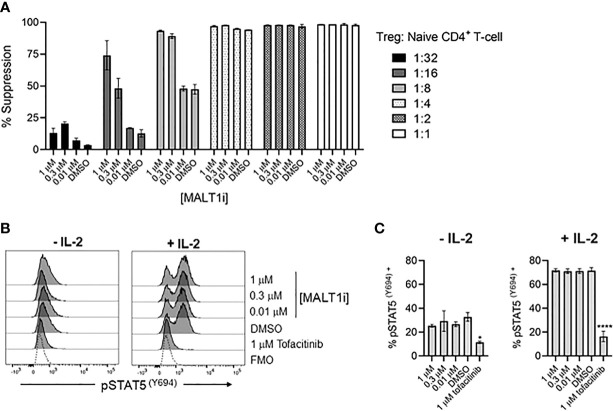
Allosteric small molecule inhibition of MALT1 does not impact human Treg function. **(A)** Percent suppression of naïve CD4^+^ T-cells by human Tregs co-cultured at indicated ratios of Tregs to naïve CD4^+^ T-cells in the presence of 1, 0.3, and 0.01 μM of MALT1i. Data is shown as the mean ± S.D. of 2 technical replicates and is representative of 2 independent experiments with 3 donors and 2 technical replicates per donor **(B, C)** Levels of pSTAT5 (Y694) were measured from Tregs pre-treated with indicated concentrations of MALT1i or 1 μM tofacitinib, followed by addition of 25 IU IL-2, presented as **(B)** histograms and **(C)** average MFIs. An unstained control is included in histograms, indicated as Fluorescence Minus One (FMO). Significant differences from DMSO-treated Tregs were calculated *via* One-way ANOVA using Graphpad Prism, *p < 0.02, ****p < 0.0001. Data is shown as the mean ± S.D. of 2 technical replicates and is representative of 3 independent experiments with 2 donors and 2-3 technical replicates per donor.

To assess whether MALT1 inhibition directly impacted Treg suppressive function, we employed an *in vitro* Treg suppression assay. Primary human Tregs from three healthy donors were co-cultured with CTV- labeled responder naïve CD4^+^ T-cells, at varying Treg: Tresponder (naïve CD4^+^ T-cells) cell ratios, and in the presence of titrated concentrations of MALT1i. MALT1 inhibition did not reduce the ability of human Tregs to suppress naïve CD4^+^ T-cell proliferation induced by CD3/CD28 stimulation using Dynabeads (**Figure 6A**, **Supplementary Figure 3A**). MALT1i also had no effect on Treg FoxP3 expression (**Supplementary Figure 3B**). At lower ratios of Tregs to responder CD4^+^ T-cells, higher compound concentrations increased the apparent suppressive capacity of Tregs. This observation likely reflects the effects of the compound on proliferation of anti-CD3/-CD28 stimulated CD4^+^ T-cells responders. Labeled responder CD4^+^ T-cells cultured with higher concentrations of MALT1i, and in the absence of co-cultured Tregs, showed decreased proliferative capacity (**Supplementary Figure 3C, D**). Overall, these data suggest that MALT1 inhibition does not directly impact the ability of Tregs to suppress naïve CD4^+^ T-cell proliferation *in vitro*, nor impact FOXP3 expression.

### Blockade of MALT1 Does Not Impact IL-2R Signaling

Since IL-2 is a primary cytokine sustaining the Treg compartment (21), we tested whether MALT1i affected IL-2R signaling. IL-2R signaling results in phosphorylation of STAT5 (pSTAT5) and is dependent on JAK1/3, so we used a flow cytometric readout of pSTAT5 to measure IL-2R activity and used the JAK1/3-inhibitor tofacitinib as a positive control for inhibition of pSTAT5. Human Tregs were pre-treated with indicated concentrations of MALT1i or tofacitinib, and pSTAT5 levels were measured in rested and IL-2-stimulated cells. Interestingly, Tregs appeared to have elevated basal levels of pSTAT5 that were reduced with tofacitinib treatment. IL-2 treatment increased pSTAT5 levels in all conditions except for the tofacitinib-treated group. Importantly, no effects of MALT1i were observed on either basal or IL-2 treated cells, suggesting that MALT1 does not play an important role in this pathway (**Figures 6B, C**), and that chronic inhibition of MALT1 spares IL-2R-dependent signaling in Tregs.

## Discussion

MALT1 has a central role in mediating immunoreceptor signaling across a range of immune and non-immune cell types. The importance of MALT1 in regulating immune cell activation is supported by reports showing that genetic and pharmacologic inhibition of MALT1 can protect against and ameliorate inflammatory processes in preclinical models (12). In addition to regulating immune effector cell responses, MALT1 signaling has also been shown to support the development and maintenance of a replete and functional Treg compartment (13, 22), which is required for immunological homeostasis. The relative contribution of MALT1 signaling to pro-inflammatory cell activation compared to Treg maintenance has not been formally addressed in the context of inflammation. MALT1 inhibitors are currently being investigated in a handful of early phase clinical trials (e.g., ClinicalTrials.gov identifier: NCT04876092, NCT04859777, NCT05144347) in solid and hematological cancers, however, their therapeutic potential in autoimmune inflammatory diseases remains untested. The results reported herein uncover for the first time a viable path for developing MALT1 inhibitors for the treatment of A&ID.

The data presented here demonstrate that MALT1 inhibition is effective in interfering with both systemic and tissue-specific pathogenic drivers of disease. MALT1i significantly blocked IL-6 expression in the plasma of rats with CIA at the highest dose tested, and expression of KC-GRO, the rodent ortholog of IL-8, at all doses tested. A similar effect was observed in the synovium with KC-GRO and TNFα, where both factors were significantly inhibited at all doses of MALT1i tested. The local tissue and systemic effects on KC-GRO are consistent with the impact of MALT1 inhibition on IL-17 production, which induces KC-GRO expression and then, in turn, recruits neutrophils to the site of inflammation (23). These data are consistent with the published effects on MALT1 inhibition on antibody dependent arthritic responses where neutrophil infiltrates were reduced (14) and are consistent with data in our study and others on the *in vitro* effects of MALT1 loss or inhibition on immune cell effector function *in vitro* (16, 19).

The anti-inflammatory action of MALT1 inhibition across multiple immune cell types and pro-inflammatory mechanisms makes MALT1 a compelling target for the treatment of autoimmune and inflammatory disease. However, the therapeutic potential of targeting MALT1 has been hindered by concerns about the impact of MALT1 inhibition on Treg homeostasis. Consistent with earlier reports, we observed reduction in CD25^+^FoxP3^+^ Tregs in the rat CIA model following treatment with MALT1i. However, when compared with naïve animals, the decrease was significant only in the group receiving the highest dose. On the contrary, disease scores and concentrations of proinflammatory cytokine/chemokine in plasma and synovial fluid were significantly reduced at all doses tested. Compound exposure-dependent uncoupling of efficacy from Treg reduction is observed following treatment with additional, structurally distinct inhibitors indicating this phenomenon of uncoupling is a generalizable feature of pharmacological modulation of MALT1 and not a compound specific feature. Furthermore, we also demonstrate increased sensitivity of Tregs to MALT1 inhibitor treatment in naïve animals when compared to rats with CIA. It should be noted that previous toxicology studies with MALT1i effects on Tregs and inflammation were performed in naïve, non-diseased animals and concentrations exceeding the IC_90_ for the compound (14). These findings present a path forward for an allosteric MALT1i for the treatment of autoimmune inflammatory diseases. Namely, the relative sensitivity of Treg reduction in the context of inflammation may expand the therapeutic index beyond what has been observed in non-diseased, naive animals (14).

There may be different mechanistic explanations for the uncoupling of efficacy and Treg reduction that also support a path forward for clinical development with a MALT1 inhibitor. The inhibition of IL-2 can at least in part explain the effect of MALT1 inhibition on Treg levels but not function and could be used as a marker for target engagement as it is tightly linked to MALT1 signaling. This latter attribute qualifies it as not just a marker for target engagement but also as a proximal pharmacodynamic event following modulation of the target, as suggested in previous reports (24). In our studies, the amount of target coverage needed for efficacy with MALT1i was equivalent to, or less than, the IL-2 IC_90_ over the 24 h dosing period. Our analyses of target coverage for multiple other compounds indicate that the time exceeding the IC_90_ over the dosing period to achieve efficacy ranged from 4 to 12 h (data not shown). The ability of MALT1i to impact multiple pathogenic factors (e.g., TNFα, IL-6, KC-GRO) may underlie the observed efficacy in the absence of a full target coverage over a 24 h period. Thus, the resulting additive effect may translate to a requirement for less target coverage (e.g., IC_50_ of multiple cellular effects) to achieve a significant effect on disease score. By contrast, the impact on Tregs could be due to inhibition of only a single factor, such as IL-2, which may not be completely affected at efficacious doses. Consistent with this hypothesis is the observation that reduction in Treg numbers required target coverage exceeding the IL-2 IC_90_ for the full dosing period, regardless of the compound used. When the systemic drug concentration exceeds the IC_90_ levels for an extended period of time, e.g., >24 h, IL-2 may become limiting and impact Treg maintenance. Thus, a dosing paradigm where MALT1 inhibitor drug concentrations that do not exceed the IC_90_ for the full dosing period can be employed to widen the therapeutic window and achieve clinical benefit without impacting Treg numbers.

In summary, the data presented here supports the concept that informed dosing strategies can be used with allosteric MALT1 inhibitors to achieve efficacy without impacting Treg numbers. This could enable chronic dosing in autoimmune and inflammatory conditions and will need to be confirmed in carefully monitored clinical trials in diseased patients. As a consequence, MALT1 remains a compelling target for the treatment of autoimmunity and inflammation.

## Data Availability Statement

The original contributions presented in the study are included in the article/**Supplementary Material**. Further inquiries can be directed to the corresponding authors.

## Ethics Statement

The animal study was reviewed and approved by Animal Care and Use Committee at WuXi AppTec and Chempartner, Shanghai, China.

## Author Contributions

AL, DR, and JM conceptualized the work. *In vitro* and *in vivo* studies were designed and performed/managed by SB and JD. AC, YH, DC, and LM performed the *in vitro* experiments. Compound synthesis and biochemical characterization was performed BD and BV. Manuscript was written by SB and AL with scientific inputs from JD, JM, and DR. All authors reviewed and approved the final version of the manuscript.

## Conflict of Interest

All authors are employees and/or shareholders of Rheos Medicines.

The authors declare that this study received funding from Rheos Medicines. The funder had the following involvement with the study: study design, collection, analysis, interpretation of data, the writing of this article and the decision to submit it for publication.

## Publisher’s Note

All claims expressed in this article are solely those of the authors and do not necessarily represent those of their affiliated organizations, or those of the publisher, the editors and the reviewers. Any product that may be evaluated in this article, or claim that may be made by its manufacturer, is not guaranteed or endorsed by the publisher.
