# Supplementary material for: Pharmacological Inhibition of MALT1 Ameliorates Autoimmune Pathogenesis and Can Be Uncoupled From Effects on Regulatory T-Cells
Source: Front Immunol. 2022 May 9;13:875320. doi: 10.3389/fimmu.2022.875320 (PMC9125252; doi:10.3389/fimmu.2022.875320)

SUPPLEMENTARY METHODS

MALT1 Biochemical Assay

Inhibitor potency was evaluated by measuring enzymatic activity of full length MALT1 at varying concentrations of compound. The enzymatic assay consists of a single substrate reaction that monitors the release of a fluorescent dye upon cleavage of the peptide substrate. The peptide substrate has the following sequence: Ac-Leu-Arg-Ser-Arg-Rh110-dPro (custom synthesis from WuXi AppTec, Shanghai, China). The assay buffer consists of 50 mM HEPES, pH 7.5, 0.8 M sodium citrate, 1 mM DTT, 0.004% tween-20, and 0.005% bovine serum albumin (BSA). Steady-state kinetic analysis of peptide substrate binding resulted in a Michaelis-Menten constant (K_M) of 150 µM. The assay was performed in a 384-well F-bottom polypropylene, black microplate (Greiner Bio_One, Catalog no. 781209) at 15 nM enzyme and 30 µM peptide substrate. The reaction was quenched after 60 min with the addition of iodoacetate at a final concentration of 10 mM. Total fluorescence was measured using an Envision (PerkinElmer) with fluorescence excitation at 485 nm and emission at 520 nm.

For potency determination, 1 µL of serially diluted compound (in 100% DMSO) was pre-incubated with 40 µL of enzyme for 30 min. The reaction was initiated with the addition of 10 µL of peptide substrate. The relative fluorescence units were transformed to percent inhibition by using 0% and 100% inhibition controls as reference. The 100% inhibition control consisted of 1 µM final concentration of (S)-1-(5-chloro-6-(2H-1,2,3-triazol-2-yl) pyridin-3-yl)-3-(2-chloro-7-(1-methoxyethyl) pyrazolo[1,5-a] pyrimidin-6-yl) urea (IC_{50} = 15 nM), while the 0% inhibition control consisted of 2% DMSO. IC_{50} values were calculated by fitting the concentration-response curves to a four-parameter logistic equation in GraphPad Prism.

Immunoblotting Analysis of MALT1 Function

Primary human $CD4^+$ T cells were isolated from human donor PBMCs by negative selection (EasySep Human $CD4^+$ T cell isolation kit, Stemcell Technologies). T cells were pre-incubated with 0-30 μM of MALT1i, or DMSO as a control, for 30 min prior to stimulation with 50 ng/mL phorbol 12-myristate 13-acetate (Sigma) and 1.34 mM ionomycin (Sigma). Whole cell lysates were prepared in lysis buffer (CellLyticMT Cell Lysis reagent (Sigma)) with HALT protease and phosphatase inhibitors (ThermoFisher Scientific) and 10 nM MG-132 (Sigma). Proteins were separated on NuPAGE 4-12% Bis-Tris denaturing gels (Invitrogen, Waltham, MA) and transferred onto nitrocellulose membranes (iBLOT2, Invitrogen). After blocking for 1 h at room temperature, blots were incubated overnight in primary antibodies, washed with PBS + 0.05% Tween-20, incubated for 2 h in secondary antibodies, washed and imaged using a LI-COR Odyssey infrared imager (LI-COR Biosciences, Lincoln, NE). Antibodies were diluted in Intercept TBS-T blocking buffer (LI-COR). Bands were quantified using ImageStudio software (LI-COR Biosciences). Primary antibodies used were: anti-HOIL-1 (Millipore Sigma, MABC576), anti-BCL10 (Abcam, 33905), anti-pIKKαβ (Cell Signaling Technologies, 2078), anti-pJNK (Cell Signaling Technologies, 4668), anti-COX-IV (Cell Signaling Technologies, 4668). Secondary antibodies used were: anti-mouse IgG H+L DyLight 680 (Cell Signaling Technologies, 5470), anti-rabbit IgG H+L DyLight 800 (Cell Signaling Technologies, 5151).

SUPPLEMENTARY FIGURE LEGEND

SUPPLEMENTARY FIGURE 1. Characterization of MALT1i effects on paracaspase and scaffold functions. **(A)** Structure of MALT1i used in these studies (previously described in Martin et al, 2020). **(B)** Concentration-dependent impact on protease activity by MALT1i-dependent cleavage of HOIL-1 substrate as determined by immunoblotting. Shaded area corresponds to concentration of MALT1i necessary to achieve 50-90% target coverage as determined from the human whole blood assay (see Table 2).

SUPPLEMENTARY FIGURE 2. Flow cytometry based **i**mmunophenotyping of non-Treg cells in rat splenocytes. **(A)** Gating strategy employed to identify various immune cell subsets is shown. **(B)** Frequency of various immune cell subsets (as indicated on each plot) across different treatment groups are shown. Data plotted as mean ± S.E.M for each treatment group (n = 8). Significant difference from the vehicle treated group was calculated via One -way ANOVA using Graphpad Prism, *p<0.05, **p<0.01, ***p<0.001, ****p<0.0001.

SUPPLEMENTARY FIGURE 3. Representative flow cytometry-based characterization of the *in vitro* Treg suppression of $CD4^+$ T-cell proliferation. **(A)** CTV traces of naïve CD4 T-cells co-cultured in various Treg:naïve $CD4^+$ T-cell ratios in the presence of 1, 0.3, and 0.01 μM of MALT1i. **(B)** Histogram representation of FOXP3 MFI from Tregs stimulated with Dynabeads from 3 human donors across 2 independent experiments. **(C)** CTV traces of naïve $CD4^+$ T-cells activated with Dynabeads for 3 days in culture in the presence of 1, 0.3, and 0.01 μM of MALT1i. **(D)** Quantitation (mean ± S.D.) of percent proliferating naïve CD4 T-cells from data represented in (C). Significant differences from the DMSO-treated group were calculated using One-way ANOVA using Graphpad Prism, **p<0.01. **(E)** Quantitation (mean ± S.D.) of the percent CD25

positive naïve $CD4^+$ T-cells when cultured alone or co-cultured in various Treg:naïve $CD4^+$ T-cell ratios in the presence of 1, 0.3, and 0.01 μM of MALT1i. Data is representative of 2 independent experiments with 3 donors and 2 technical replicates per donor.

SUPPLEMENTARY FIGURE 1

A

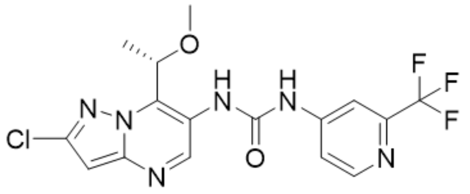

MALT1i

B

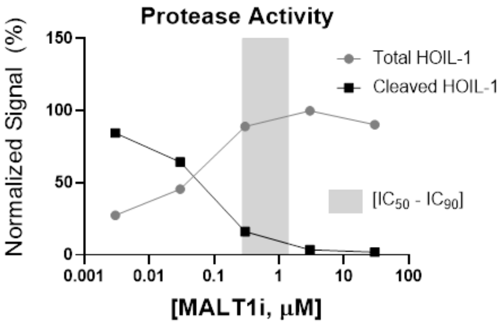

SUPPLEMENTARY FIGURE 2

A

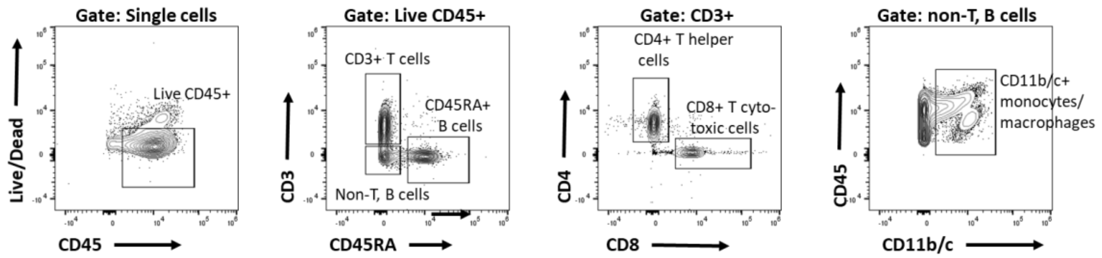

B

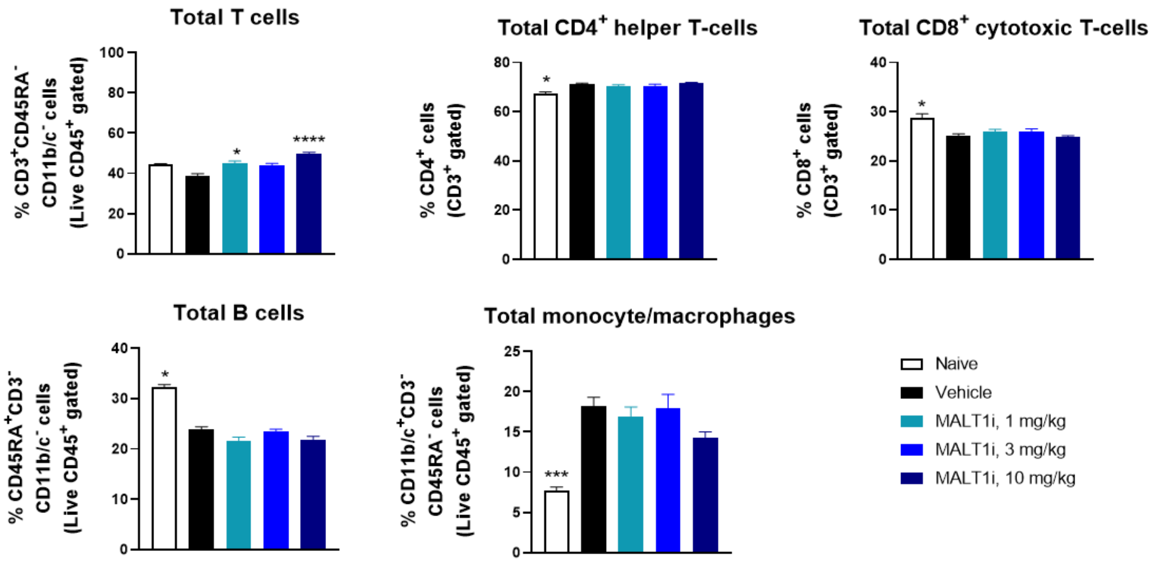

SUPPLEMENTARY FIGURE 3

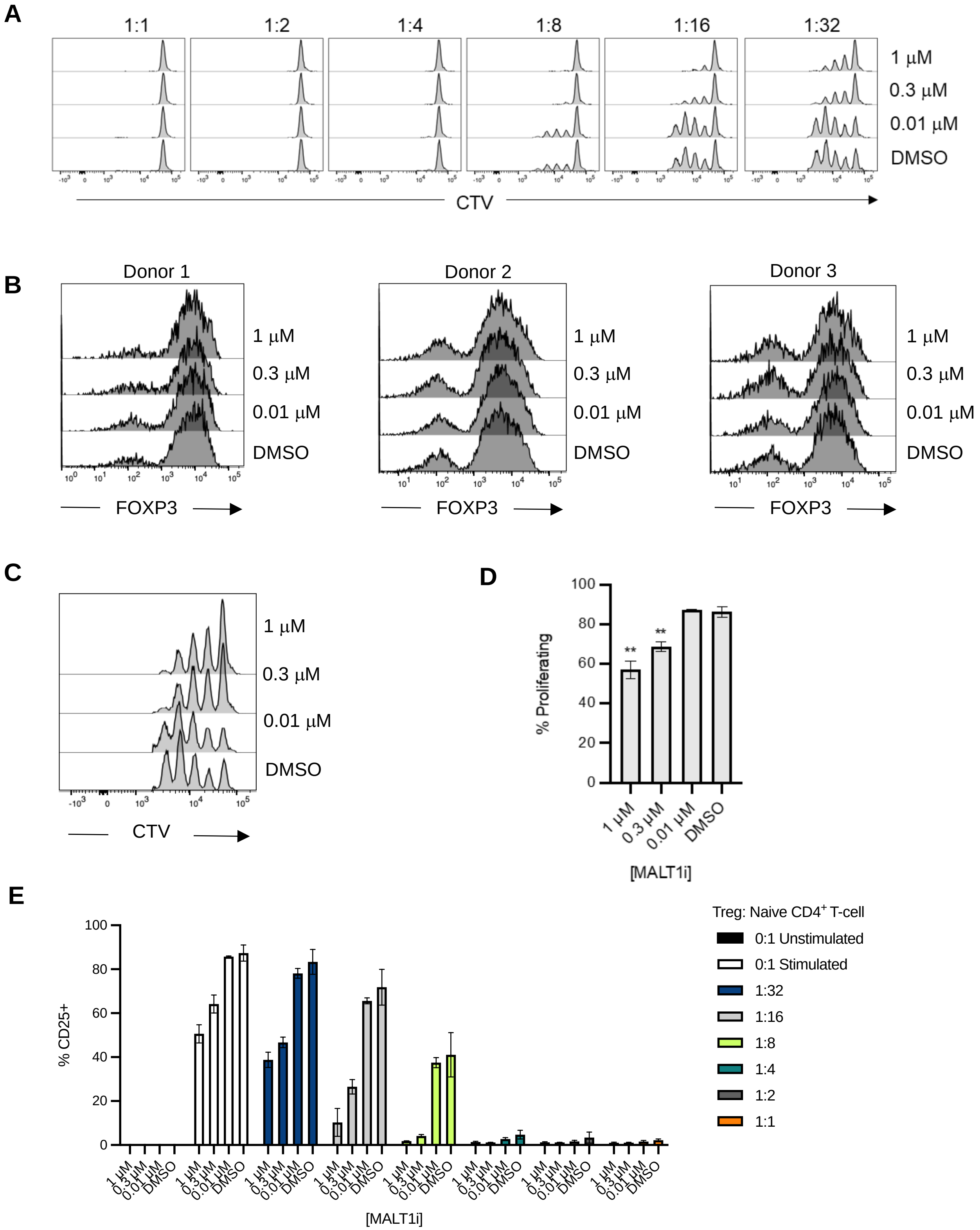

Supplement: Supplementary file 1 [file DataSheet_1.pdf]
